# Residual Stress in Lithium Niobate Film Layer of LNOI/Si Hybrid Wafer Fabricated Using Low-Temperature Bonding Method

**DOI:** 10.3390/mi10020136

**Published:** 2019-02-18

**Authors:** Ryo Takigawa, Toru Tomimatsu, Eiji Higurashi, Tanemasa Asano

**Affiliations:** 1 Graduate School of Information Science and Electrical Engineering, Kyushu University, 744 Motooka, Nishi-ku, Fukuoka 819-0395, Japan; asano@ed.kyushu-u.ac.jp; 2 Graduate School of Science, Tohoku University, 6-3 Aramaki Aza Aoba, Aobaku, Sendai 980-8578, Japan; tomimatu@m.tohoku.ac.jp; 3 Research Center for Ubiquitous MEMS and Micro Engineering, National Institute of Advanced Industrial Science and Technology (AIST), 1-2-1 Namiki, Tsukuba, Ibaraki 305-8564, Japan; 4 Department of Precision Engineering, School of Engineering, The University of Tokyo, 7-3-1 Hongo, Bunkyo-ku, Tokyo 153-8904, Japan; eiji@su.t.u-tokyo.ac.jp

**Keywords:** Residual stress in LN film layer, Lithium niobate-on-insulator/Si hybrid wafer, Surface-activated bonding, Low-temperature wafer bonding, Large mismatch of thermal expansion coefficient

## Abstract

This paper focuses on the residual stress in a lithium niobate (LN) film layer of a LN-on-insulator (LNOI)/Si hybrid wafer. This stress originates from a large mismatch between the thermal expansion coefficients of the layers. A modified surface-activated bonding method achieved fabrication of a thin-film LNOI/Si hybrid wafer. This low-temperature bonding method at 100 °C showed a strong bond between the LN and SiO_2_ layers, which is sufficient to withstand the wafer thinning to a LN thickness of approximately 5 μm using conventional mechanical polishing. Using micro-Raman spectroscopy, the residual stress in the bonded LN film in this trilayered (LN/SiO_2_/Si) structure was investigated. The measured residual tensile stress in the LN film layer was approximately 155 MPa, which was similar to the value calculated by stress analysis. This study will be useful for the development of various hetero-integrated LN micro-devices, including silicon-based, LNOI-integrated photonic devices.

## 1. Introduction

Lithium niobate (LiNbO_3_: LN) is a unique ferroelectric material with excellent non-linear optical, electro-optical, piezo-electrical, and pyro-electrical characteristics. It has numerous applications, including frequency convertors, ultra-fast optical modulators, surface acoustic wave filters, and pyro-imaging sensors. Recently, thin-film LN-on-insulator (LNOI) wafer technology has revolutionized the fabrication of LN devices and created a future platform that can fully exploit the unique properties of LN [1,2]. For example, LNOI waveguides have received significant attention for photonics applications [3,4,5,6]. Compared with conventional LN waveguides, high refractive index contrast (HRIC) LNOI waveguides exhibit a much stronger confinement, which results in a smaller device with improved performance. The integration of an LNOI device with a promising Si platform, which currently is utilized for optical benches, microelectromechanical systems, large-scale integration, and Si photonics, has the potential for producing next-generation, highly functional microsystems [5,6,7]. To this end, the direct bonding of LN and SiO_2_/Si wafers is a powerful approach.

To date, direct bonding of LN and Si wafers has been challenging because of a large mismatch in the coefficient of thermal expansion (CTE) for these two materials. Therefore, low-temperature bonding is essential for overcoming this serious CTE mismatch. Our previous research suggested that the post-bonding annealing temperature of a directly bonded LN/Si wafer should be limited to approximately 100 °C [8,9,10]. Conventional plasma-activated bonding has been employed for the direct bonding of LN and Si [11], but annealing (usually performed above 200 °C for a few hours [12]) is required for the bonded wafers to withstand the post-bond, wafer-thinning processing, which uses conventional mechanical polishing required for waveguide fabrication.

A promising, low-temperature alternative for heterogeneous integration is surface-activated bonding (SAB) [8,9,13,14,15,16,17]. However, previous research has demonstrated via experiment that it is difficult to apply the conventional SAB method to the direct bonding of oxide materials, such as SiO_2_ [18]. To overcome this limitation, a modified SAB method has been proposed [19,20,21,22]. During the SAB process, the deposition of the Fe adhesion layer on each wafer is deployed simultaneously. To date, this modified SAB demonstrated room temperature bonding of LN-Si, SiO_2_-SiO_2_, and SiO_2_-SiN. We applied this method using an ultrathin film of Fe to room temperature bonding of LN and SiO_2_ to minimize the propagation loss through a LNOI waveguide due to large absorption of Fe [23,24]. However, it is difficult to obtain sufficient bond strength at room temperature to withstand the above-mentioned post-bond wafer-thinning processing. Annealing is one effective approach to improve bond strength. Annealing leads to residual stress in the bonded LN which has resulted from the large CTE mismatch. Mismatch also causes a change to the refractive index, which is important in photonics applications such as the waveguide-type ring resonator. Although room temperature bonding using sputtered Si film as the adhesion layer was also demonstrated [22,25,26,27], the application to photonic devices, including thin-film LNOI waveguides, was limited because the Si film was transparent in only a section of the infrared region.

This study focused on the residual stress in an LN film layer bonded to a SiO_2_/Si wafer. We used a modified SAB method, which combined a Fe ultrathin film with annealing to form a strong bond between LN and Si thermal oxidized film (SiO_2_). This bond is sufficient to withstand post-bond wafer-thinning that uses conventional mechanical polishing and is necessary for future applications, such as HRIC waveguide fabrication. The residual stress in the bonded LN film layer, which originated from the large CTE mismatch in the trilayered (LN/SiO_2_/Si) system, was measured by micro-Raman spectroscopy. The residual stress state was also investigated numerically. 

## 2. Experimental Procedure

**Figure 1:**
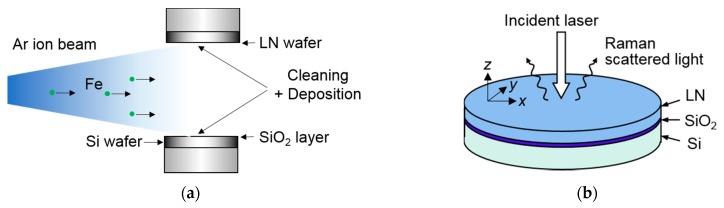
(**a**) Schematic diagram of modified surface activation process. (**b**) Schematic illustration of measurement of stress in bonded lithium niobate (LN) film layer.

Three-inch, double-polished, single-crystalline LN (Z-cut) and Si wafers were purchased, and a 1-μm-thick SiO_2_ layer was thermally grown on the Si wafer. The thicknesses of the LN and Si wafers were 500 μm and 360 μm, respectively. The modified SAB process used Ar ion beam bombardment to bond the negative surface of the LN wafer to the SiO_2_ layer on the Si wafer. During the Ar ion beam bombardment, both surface cleaning and deposition of an ultrathin Fe layer were deployed simultaneously on both the negative surface of the LN wafer and the SiO_2_ surface, as shown in Figure 1a. 

It was intended that a large number of Fe atoms would emerge from the stainless steel ion source during the bombardment. Each wafer was placed in the bonding chamber; the chamber was then evacuated. The bombardment was performed when the background vacuum pressure dropped below 1 × 10^−5^ Pa, after which the surfaces were contacted with an applied load of approximately 22 MPa at room temperature. The ion beam bombardment time was 4 min, which is a condition sufficient for removing the organic contaminants on the bonding surface. Subsequently, the bonded wafers at room temperature were annealed at 100 °C in ambient air. Finally, the thickness of the bonded LN wafer was reduced by conventional mechanical polishing to complete the fabrication of a thin-film LNOI/Si hybrid wafer. The bond quality was evaluated using a dicing test and high-resolution transmission electron microscopy (TEM). The residual stress in the bonded LN film layer was evaluated by micro-Raman spectroscopy, as shown in Figure 1b. The stress measurement was conducted at room temperature in ambient air. The light source for the incident laser beam was operated at a wavelength of 514.5 nm. The laser illuminated the top surface of LN film layer and was focused on the interface between LN and SiO_2_.

## 3. Results and Discussion

### 3.1. Thin-Film LNOI/Si Hybrid Wafer

**Figure 2:**
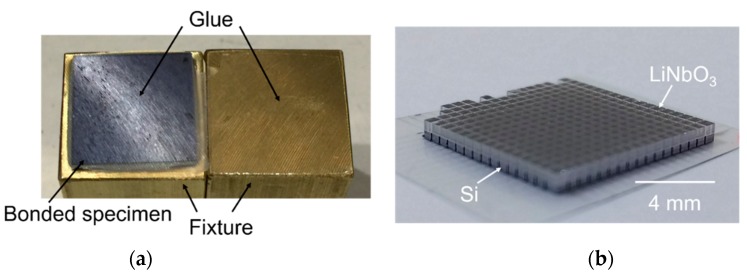
(**a**) Image of the bonded specimen fracture during tensile test. (**b**) Image of the diced 0.5 mm × 0.5 mm chips.

Nearly void-free wafer bonding of LN and SiO_2_/Si was demonstrated without serious crack generation [23]. Pulling tensile tests and dicing tests were performed to evaluate the strength of the wafer bond. The bonded wafer was diced into 10 mm × 10 mm chips, and these were glued to the fixtures of a pulling test machine. The tensile strength was measured as approximately 10 MPa. After the tensile test, we confirmed that peeling occurred between the pulling test fixture and adhesive glue, as shown in Figure 2a. This indicates that the bond between the LN and SiO_2_ bonding interface was stronger than the glue. Next, 10 mm × 10 mm chips were diced into 0.5 mm × 0.5 mm chips using a dicing saw. This test produced perfect diced chips without any interfacial de-bonding, as shown in Figure 2b, indicating that a strong bond was formed between LN and SiO_2._

**Figure 3:**
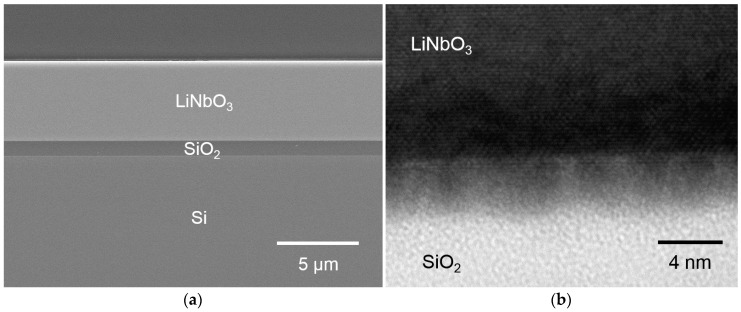
(**a**) Cross-sectional scanning electron microscopy (SEM) image of the LNOI/Si hybrid wafer. (**b**) Cross-sectional transmission electron microscopy (TEM) image of the LN/SiO_2_ bonding interface.

The thickness of the bonded LN wafer was reduced from 500 μm to less than 5 μm, which is required for HRIC waveguide fabrication. Figure 3a shows cross-sectional, scanning electron microscopy (SEM) images of the resulting LNOI/Si wafer. No peeling was observed throughout the bonding interface, which indicated that the bond between LN and SiO_2_ was sufficiently strong to withstand wafer thinning via mechanical polishing. The nanostructure of the bonding interface was also observed by TEM. Figure 3b shows a cross-sectional TEM image of the bonding interface, which indicates that no cracks or voids existed at the bonding interface at the nanoscale level. Atomic-level close contact between the LN and SiO_2_ was confirmed. In addition, the Ar ion beam bombardment appeared not to have damaged the single crystalline LN wafer because the lattice fringe could be seen. Observations of the bonding interface indicated that the bond was strong enough to withstand wafer thinning by mechanical polishing. As shown, we could observe an approximately 4-nm-thick uniform intermediate layer, which was formed during surface activation and low-temperature annealing processes. Per previous research [24], the thickness of the Fe-containing layer deposited under this condition had a thickness of a few nanometers. The diffusion created by this low-temperature annealing process may have enhanced the bond strength between LN and SiO_2._

### 3.2. Residual Stress in LN Film Layer

**Figure 4:**
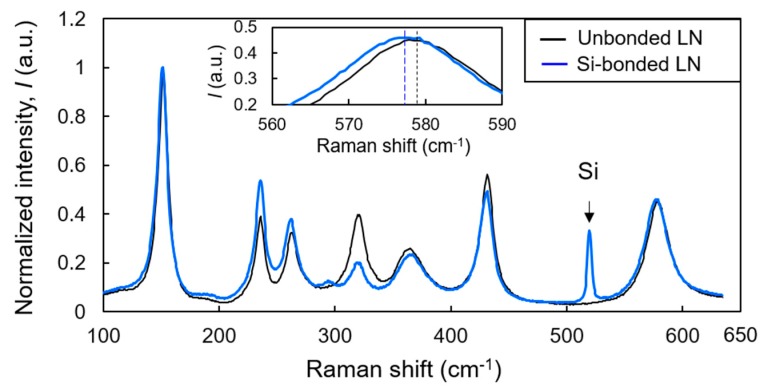
Raman spectra of unbonded LN and Si-bonded LN. Intensity is normalized to the maximum intensity of the 152 cm^−1^ line. Enlarged spectra around 578 cm^−1^ are shown in the inset, where the peak positions are depicted by dashed lines.

To evaluate the thermal stress in an LN film layer bonded to Si, micro-Raman spectroscopy was conducted, as shown in Figure 1b. Figure 4 shows typical Raman spectra obtained from unbonded and bonded LN. Because the measurement was performed under backscattering geometry, the Raman lines of E(TO) modes (i.e., 152, 236, 263, 322, 369, 432, and 578 cm^−1^) were predominantly observed [28]. We found significant differences in the Raman intensity between samples in the range of 200–400 cm^−1^, which may have been caused by uniform lattice deformation and not by inhomogeneous defects. In previous work, the stress-induced Raman intensity change below 500 cm^−1^ was thought to be related to LN phase transition [29]. In this study, however, the LN stress (discussed below) was much lower than the phase transition stress; thus, we can assume that bonded and unbonded LN have the same structure. In this frequency region (200–400 cm^−1^), the closely spaced lines cause seeming shifts in neighboring peaks when each line intensity changed. To rule out this unwanted peak shift for stress estimation, we focused on the line at 578 cm^−1^, away from the closely spaced line region; the line position at 578 cm^−1^ was not disturbed by neighboring peaks. Because physical properties of Si and SiO_2_ layers are isotropic, and the LN principal axis of the triagonal crystallographic system is normal to the bonded surface, stress stays in an equi-biaxial state (i.e., σ_x_ = σ_y_ = σ, σ_z_ = 0) near the center of the sample under the assumption of planar stress, in which x and y are lateral directions, and z is the direction of thickness. By defining the LN crystallographic coordinates to coinciding axes with laboratory coordinates, the lateral stress σ is given by

(1) $$\sigma =\frac{\Delta \omega }{2\left[a_{E}(S_{11}+S_{12}\right)+b_{E}S_{13}]}$$

where Δ*ω* is the Raman line shift with respect to unbonded LN, *a*_E_ and *b*_E_ are phonon deformation potential parameters (*a*_E_ = −900 cm^−1^ and *b*_E_ = −50 cm^−1^ for the line at 578 cm^−1^), and *S_ij_* are the elastic compliance constants (*S*_11_ = 5.78 × 10^−12^ Pa^−1^, *S*_12_ = −1.01 × 10^−12^ Pa^−1^, and *S*_13_ = −1.47 × 10^−12^ Pa^−1^) [30]. Because the deviation of the peak position of bonded LN from that of unbonded LN is represented as Δ*ω* = −1.38 cm^−1^, we estimated the lateral tensile stress to be *σ* ≈ 155 MPa.

Next, the calculation of the residual stress in the bonded LN film layer was performed. Because the thicknesses of the LN and SiO_2_ layers are considerably smaller than the thickness of Si, the stress gradient induced by the warpage in the trilayered system can be negligible, according to a multilayer model that considers the bending effect [31]. Therefore, we focused on the uniform component of the lateral stress in the LN layer, σ_LN_, described by the following equation [31]:

(2) $$\sigma_{\mathrm{LN}}=-\frac{E_{\mathrm{LN}}\Delta T\left[E_{{\mathrm{SiO}}_{2}}h_{{\mathrm{SiO}}_{2}}\left(\alpha_{\mathrm{LN}}-\alpha_{{\mathrm{SiO}}_{2}}\right)+E_{\mathrm{Si}}h_{\mathrm{Si}}\left(\alpha_{\mathrm{LN}}-\alpha_{\mathrm{Si}}\right)\right]}{E_{\mathrm{LN}}h_{\mathrm{LN}}+E_{{\mathrm{SiO}}_{2}}h_{{\mathrm{SiO}}_{2}}+E_{\mathrm{Si}}h_{\mathrm{Si}}}$$

where *E*_LN_, *E*_SiO2_, and *E*_Si_ denote the biaxial elastic moduli of LN, SiO_2_, and Si, respectively; *h*_LN_, *h*_SiO2_, and *h*_Si_ are the thicknesses of the LN, SiO_2_, and Si layers, respectively; and T is temperature. Thermal stress in the model becomes biaxial and the solution can be obtained by replacing Young’s modulus, E’, with the biaxial elastic modulus, E, where E = E’/(1−v) and v is Poisson’s ratio. The residual stress was calculated under the conditions that the LN and Si wafers were bonded at 100 °C and then cooled to room temperature (25 °C) without debonding. Although LN is known to be highly anisotropic, Si is assumed to be isotropic. $E_{\mathrm{LN}}$ can be estimated using the following equation:

(3) $$E_{\mathrm{LN}}=C_{11}+C_{12}-\frac{2({C_{13}}^{2})}{C_{33}}$$

The following parameters were used for the calculation: elastic stiffness coefficients of LN = 203 GPa (*C*_11_), 57.3 GPa (*C*_12_), 75.2 GPa (*C*_13_), and 242.4 GPa (*C*_33_) [32]. Using Equation (3), $E_{\mathrm{LN}}$ was calculated to be 213.6 GPa. The thermoelastic properties used in the calculation of residual stress are summarized in Table 1. With Equation (2), the tensile stress in the LN film layer was estimated to be approximately 170 MPa, which is similar to and validates the value obtained by the experimental results using micro-Raman spectroscopy. Stresses (σ) in the LN layer were almost constant along the lateral (x and y) direction in the area away from the wafer edge because the total thickness of the hybrid wafer was negligibly small compared to the wafer diameter. In addition, the stress gradient inside the LN layer, arising from warpage, was suppressed as a result of the small thickness ratio of LN to Si (~1%); the stress variation in LN induced by the bending due to thermal stress was less than 0.1% of σ. Thus, the center area of the LN layer was subjected to a nearly homogeneous stress of ~155 MPa. Such homogeneity of the LN stress gives us a unique stress criterion for inspecting the bonding durability of LN, which was demonstrated to be appropriately assessed by Raman spectroscopy in this study. This study will be useful for the development of LNOI waveguide devices, including periodically poled waveguide devices and micro ring-resonators, because the refractive index of LN crystal tends to change on account of residual stress. Further investigation into the fabrication process, as well as systematic stress measurement with different annealing temperatures, would provide physical insights into the optical properties of bonded LN film, the strong bond condition, and the fracture mechanism of the hybrid structure.

## 4. Conclusions

In this study, we investigated the residual stress that results when an LN film layer is bonded to a SiO_2_/Si wafer using modified low-temperature SAB with annealing at 100 °C. This low-temperature bonding method produced a bond between LN and SiO_2_ that was strong enough to withstand wafer thinning (down to an LN thickness of approximately 5 μm) using conventional mechanical polishing. Micro-Raman spectroscopy was used to estimate the residual stress in the bonded LN film layer produced during this work as approximately 155 MPa, which is similar to the value obtained by stress analysis calculations. Although the experimental results show large tensile residual stress in the bonded LN film layer, the thin-film LNOI/Si hybrid wafer was not fractured at a tensile residual strength up to 155 MPa. This result is specifically useful for the bonding of LN thin films but also for the bonding of other dissimilar materials with a large CTE mismatch. The SAB method produced a bond suitable for post-bond processing for device fabrication. We believe that this study contributes to the development of future hetero-integrated LN micro-devices, including thin-film LNOI waveguide circuits with a strong optical confinement on Si platforms, thin-film surface acoustic wave filters, and pyro-imaging sensors. 

## Figures and Tables

**Table 1:** Material parameters used in the calculation [33,34,35,36].

Material	CTE (× 10^−6^/K)	Biaxial Elastic Modulus (GPa)	Thickness (μm)
LN	13.4	213.6	5
SiO_2_	1.0	77	1
Si	2.6	180.6	360

## References

[1] Poberaj G., Hu H., Sohler W., Guenter P. (2012). Lithium niobate on insulator (LNOI) for microphotonics devices. Laser Photon. Rev..

[2] Boes A., Corcoran B., Chang L., Bowers J., Mitchell A. (2018). Status and Potential of Lithium Niobate on Insulator (LNOI) for Photon Integrated Circuits. Laser Photon. Rev..

[3] Rabiei P., Gunter P. (2004). Optical and electro-optical properties of submicrometer lithium niobate slab waveguides prepared by crystal ion slicing and wafer bonding. Appl. Phys. Lett..

[4] Guarino A., Poberaj G., Rezzonico D., Degl’Innocenti R., Gunter P. (2007). Electro-optically tunable microring resonators. Nat. Photon..

[5] Mercante A.J., Yao P., Shi S., Schneider G., Murakowski J., Prather D.W. (2016). 110 GHz CMOS compatible thin film LiNbO_3_ modulator on Silicon. Opt. Express.

[6] Wang C., Zhang M., Chen X., Bertrand M., Ansari A.S., Chandrasekher S., Wintzer P., Loncar M. (2018). Integrated lithium niobate electro-optic modulators operating at CMOS-compatible voltage. Nature.

[7] Park Y.B., Min B., Vahala K.J., Atwater H.A. (2006). Integration of single-crystal LiNbO_3_ thin film on silicon by laser irradiation and ion implantation-induced layer transfer. Adv. Mater..

[8] Takagi H., Maeda R., Suga T. (2001). Room-temperature wafer bonding of Si to LiNbO_3_, LiTaO_3_ and Gd_3_Ga_5_O_12_ by Ar-beam surface activation. J. Micromech. Microeng..

[9] Takigawa R., Higurashi E., Suga T., Shinada S., Kawanishi T. (2007). Low-temperature Au-Au bonding for LiNbO_3_/Si structure achieved in ambient air. IEICE Trans. Electron..

[10] Takigawa R., Kawano H., Ikenoue H., Asano T. (2017). Investigation of the interface between LiNbO_3_ and Si wafers bonded by laser irradiation. Jpn. J. Appl. Phys..

[11] Wu C.C., Horng R.H., Wuu D.S., Chen T.N., Ho S.S., Ting C.J., Tsai H.Y. (2006). Thinning technology for lithium niobate wafer by surface activated bonding and chemical mechanical polishing. Jpn. J. App. Phys..

[12] Pasquariello D., Hjort K. (2002). Plasma-assisted InP-to-Si low temperature wafer bonding. IEEE J. Sel. Top. Quant. Electron..

[13] Takagi H., Maeda R., Hosoda N., Suga T. (1999). Room-temperature bonding of lithium niobate and silicon wafers by argon-beam surface activation. Appl. Phys. Lett..

[14] Takigawa R., Higurashi E., Suga T., Sawada R. (2008). Room-temperature bonding of vertical-cavity surface-emitting laser diode chips on Si substrates in ambient air. Appl. Phys. Exp..

[15] Takigawa R., Higurashi E., Suga T., Kawanishi T. (2011). Passive alignment and mounting of LiNbO_3_ waveguide chips on Si substrates by low-temperature solid-state bonding of Au. IEEE J. Sel. Top. Quant. Electron..

[16] Takigawa R., Higurashi E., Suga T., Kawanishi T. (2011). Air-gap structure between integrated LiNbO_3_ optical modulators and micromachined Si substrates. Opt. Express.

[17] Takigawa R., Higurashi E., Suga T., Kawanishi T. (2017). Room-temperature transfer bonding of Lithium niobate thin film on micromachined silicon substrates with Au microbumps. Sens. Actuators A..

[18] Takagi H., Maeda R., Chung T.R., Suga T. (1998). Low-temperature direct bonding of silicon and silicon oxide by the surface activation method. Sens. Actuators A.

[19] Howlader M.M.R., Okada H., Kim T.H., Itoh T., Suga T. (2004). Wafer level surface activated bonding tool for MEMS packaging. J. Electrochem. Soc..

[20] Howlader M.M.R., Suga T., Kim M.J. (2006). Room temperature bonding of silicon and lithium niobate. Appl. Phys. Lett..

[21] Kondou R., Wang C., Shigetou A., Suga T. (2012). Nanoadhesion layer for enhanced Si-Si and Si-SiN wafer bonding. Microelectron. Reliab..

[22] Suga T., Mu F., Fujino M., Takahashi Y., Nakazawa H., Iguchi K. (2015). Silicon carbide wafer bonding by modified surface activated bonding method. Jpn. J. Appl. Phys..

[23] Takigawa R., Higurashi E., Asano T. Surface activated wafer bonding of LiNbO_3_ and SiO_2_/Si for LNOI on Si. Proceedings of the 2017 5th International Workshop on Low Temperature Bonding for 3D Integration (LTB-3D).

[24] Takigawa R., Higurashi E., Asano T. (2018). Room-temperature wafer bonding of LiNbO_3_ and SiO_2_ using surface activated bonding method. Jpn. J. Appl. Phys..

[25] Shimatsu T., Uomoto M. (2010). Atomic diffusion bonding of wafers with thin nanocrystalline metal films. J. Vac. Sci. Technol. B.

[26] Utsumi J., Ide K., Ichiyanagi Y. (2016). Room-temperature bonding of SiO_2_ and SiO_2_ by surface activated bonding method using Si ultrathin films. Jpn. J. Appl. Phys..

[27] Takigawa R., Asano T. (2018). Thin-film lithium niobate-on-insulator waveguides fabricated on silicon wafer by room-temperature bonding method with silicon nanoadhesive layer. Opt. Express.

[28] Fontana M.D., Bourson P. (2015). Microstructure and defects probed by Raman spectroscopy in lithium niobate crystals and devices. Appl. Phys. Rev..

[29] Suchocki A., Paszkowicz W., Kamińska A., Durygin A., Saxena S.K., Arizmendi L., Bermudez V. (2006). Influence of stoichiometry on phase transition pressure of LiNbO_3_. Appl. Phys. Lett..

[30] Pezzotti G., Hagihara H., Zhu W. (2013). Quantitative investigation of Raman selection rules and validation of the secular equation for trigonal LiNbO_3_. J. Phys. D: Appl. Phys..

[31] Hsueh C.H., Lee S. (2003). Modeling of elastic thermal stresses in two materials joined by a graded layer. Composites: Part B.

[32] Smith R.T., Welsh F.S. (1971). Temperature Dependence of the Elastic, Piezoelectric, and Dielectric Constants of Lithium Tantalate and Lithium Niobate. J. Appl. Phys..

[33] Pignatiello F., De Rosa M., Ferraro P., Grilli S., De Natale P., Arie A., De Nicola S. (2007). Measurement of the thermal expansion coefficients of ferroelectric crystals by a Moire interferometer. Opt. Commun..

[34] Hopcroft M.A., Nix W.D., Kenny T.W. (2010). What is the young modulus of Silicon?. J. Microelectromech. Syst..

[35] Swenson C.A. (1983). Recommended values for the thermal expansivity of silicon from 0 to 1000 K. J. Phys. Chem. Ref. Data.

[36] Zhao J.H., Ryan T., Ho P.S., McKerrow A.J., Shih W.Y. (1999). Measurement of elastic modulus, Poisson ratio, and coefficient of thermal expansion of on-wafer submicron films. J. Appl. Phys..

